# Divergent T-cell receptor recognition modes of a HLA-I restricted extended tumour-associated peptide

**DOI:** 10.1038/s41467-018-03321-w

**Published:** 2018-03-12

**Authors:** Kok Fei Chan, Benjamin S. Gully, Stephanie Gras, Dennis X. Beringer, Lars Kjer-Nielsen, Jonathan Cebon, James McCluskey, Weisan Chen, Jamie Rossjohn

**Affiliations:** 1 Olivia Newton-John Cancer Research Institute, and School of Cancer Medicine, La Trobe University, Heidelberg, VIC 3084 Australia; 2 Department of Microbiology and Immunology, Peter Doherty Institute for Infection and Immunity, The University of Melbourne, Parkville, VIC 3010 Australia; 3 Infection and Immunity Program and Department of Biochemistry and Molecular Biology, Biomedicine Discovery Institute, Monash University, Clayton, VIC 3800 Australia; 4 Department of Biochemistry & Genetics, La Trobe Institute of Molecular Science, La Trobe University, Bundoora, VIC 3086 Australia; 5 Australian Research Council Centre of Excellence for Advanced Molecular Imaging, Monash University, Clayton, VIC 3800 Australia; 6 Institute of Infection and Immunity, Cardiff University School of Medicine, Heath Park, Cardiff, CF14 4XN UK

## Abstract

Human leukocyte antigen (HLA)-I molecules generally bind short peptides (8–10 amino acids), although extended HLA-I restricted peptides (>10 amino acids) can be presented to T cells. However, the function of such extended HLA-I epitopes in tumour immunity, and how they would be recognised by T-cell receptors (TCR) remains unclear. Here we show that the structures of two distinct TCRs (TRAV4^+^TRAJ21^+^-TRBV28^+^TRBJ2-3^+^ and TRAV4^**+**^TRAJ8^+^-TRBV9^+^TRBJ2**-**1^+^), originating from a polyclonal T-cell repertoire, bind to HLA-B*07:02, presenting a 13-amino-acid-long tumour-associated peptide, NY-ESO-1_60–72_. Comparison of the structures reveals that the two TCRs differentially binds NY-ESO-1_60–72_–HLA-B*07:02 complex, and induces differing extent of conformational change of the NY-ESO-1_60–72_ epitope. Accordingly, polyclonal TCR usage towards an extended HLA-I restricted tumour epitope translates to differing TCR recognition modes, whereby extensive flexibility at the TCR–pHLA-I interface engenders recognition.

## Abstract (web summary)

Human leukocyte antigen (HLA) presents peptides to activate T cells, but many aspects in the T cell receptor (TCR)/HLA interaction remain unclear. Here the authors show, via structural data, that two TCRs differentially recognize the same tumour peptide/HLA complex and induce contrasting conformation changes of the peptide.

## Introduction

Human leukocyte antigen (HLA)-I molecules are of central importance in the presentation of antigenic peptides, enabling CD8^+^ T cells to eliminate cancerous and virally infected cells. Typically, HLA-I molecules present peptides between 8 and 10 amino acids in length^1^, where the N- and C-termini of the peptide are fixed within the P2 and PΩ binding pockets of the antigen (Ag)-binding cleft, respectively. However, HLA-I molecules can present extended peptides (>10 amino acids), whereby the N- and C-termini are similarly constrained within the HLA-I molecule, forcing the central region of the peptide to bulge from the Ag-binding cleft^2^. In addition, the HLA-associated peptide repertoire may be further expanded via N-terminal extensions on presented peptides, as observed for HLA-B*57:01^3^. C-terminal protrusions have also been observed^4^ and were shown to extend out of the F pocket of the HLA-I binding groove^5^. Collectively, it has been estimated that extended peptides could comprise as much as 10% of the total HLA-I peptide repertoire^6–10^. Accordingly, it is difficult to predict a priori how extended peptides will be accommodated within the HLA-I molecule, and subsequently recognised by the T-cell receptor (TCR)^6,8,11^. Nevertheless, a number of studies have reported the importance of extended peptides in CD8^+^ T-cell-mediated immunity—mostly in the axis of viral immunity^6,12–17^.Extended peptides presented by HLA-I molecules can adopt highly dynamic conformations, thereby presenting differing energetic barriers for TCR ligation^1,18^. Extended peptides are generally considered to be challenging targets for TCR recognition owing to the dynamic nature of the central bulge^19–25^, in contrast to peptides of canonical length^26–28^. Extended peptides were frequently associated with highly biased T-cell repertoires^1,7,9,10,18,29^, thought to be resultant from conserved and HLA-I-centric TCR docking topologies^12–15,18,20,30^. Notwithstanding recent exceptions^31,32^, the majority of TCR–pHLA-I structural data available exhibits a fixed polarity, whereby the TCR α- and β-chains are positioned over the HLA-I α2 and α1-helices, respectively^1^, although how this is related to extended epitopes remained unclear. Relatively little is known regarding TCR engagement of extended peptides despite their apparent importance in tumour immunosurveillance^6,33^, with extended epitopes identified for the tumorigenic antigens CAMEL^34^, MAGE-A1^35,36^, and NY-ESO-1^17^. Presently, TCR recognition of extended epitopes has demonstrated starkly contrasting docking mechanisms. For example, one TCR docked atop the super-bulged LPEP (BZLF1_52-64_) peptide, making limited contact with the HLA-I molecule itself^12^. A subsequent study described how another TCR docked towards the N-terminal end of this bulged peptide, making more extensive contacts with the HLA-I although the peptide conformation remained unchanged^16^. Conversely, another crystal structure described TCR recognition of an 11-amino-acid peptide, where the TCR flattened the bulged peptide upon ligation^13^.

NY-ESO-1 is an immunogenic cancer-testis antigen that is spontaneously expressed on a range of melanomas and other cancers including myelomas^17,37^. A key mediator of NY-ESO-1 immunity is CD8^+^ T cells with observations of CD8^+^ T-cell infiltration correlating with NY-ESO-1 expression and inversely correlating with tumour progression in vivo^38^. NY-ESO-1 restricted T cells therefore are of great interest due to their potential use for targeted immunotherapeutic treatment of tumours. Indeed, NY-ESO-1-specific engineered T cells have been studied for therapeutic use in multiple myeloma treatment^39^. Here T cells raised against NY-ESO-1_157–165_ presented by HLA-A*02:01 were clonotyped^40^, structurally characterised^41^, and used for phage display to generate TCRs with picomolar affinity for the NY-ESO-1_157–165_ antigen^42^. The engineered T cell then formed the framework (FW) for engineered T-cell therapy, with the NY-ESO-1 restricted T cells showing targeted antitumour activity in clinical trials^39^.

In addition to the HLA-A*02:01-directed response, an immunodominant extended peptide was identified, which was presented by HLA-B*07:02^17^. To identify the principles underpinning extended peptide recognition, here we investigated TCR binding of an immunodominant NY-ESO-1 13-amino-acid peptide (APRGPHGGAASGL) derived from positions 60–72 of the cancer-testis antigen, NY-ESO-1. We examined the HLA-B*07:02-NY-ESO-1 restricted CD8^+^ T-cell repertoire, previously shown to exhibit a diverse TRBV gene repertoire in vaccinated HLA-B*07:02^+^ melanoma patients^17^. Further, we isolated and characterised four individual T-cell clones that were representative of the many TRBV families. The binding of two distinct TCRs to NY-ESO-1_60–72_-HLA-B*07:02 was via either flattening or stabilisation of the extended peptide. This represents the first example of how a HLA-restricted peptide adopts markedly differing conformations upon engaging differing TCRs, thereby providing key insights into TCR recognition of extended peptides in the context of polyclonal TCR recognition.

## Results

### NY-ESO-1_60__–72_-HLA-B*07:02-specific CD8^+^ T-cell clonotypes

**Fig. 1:**
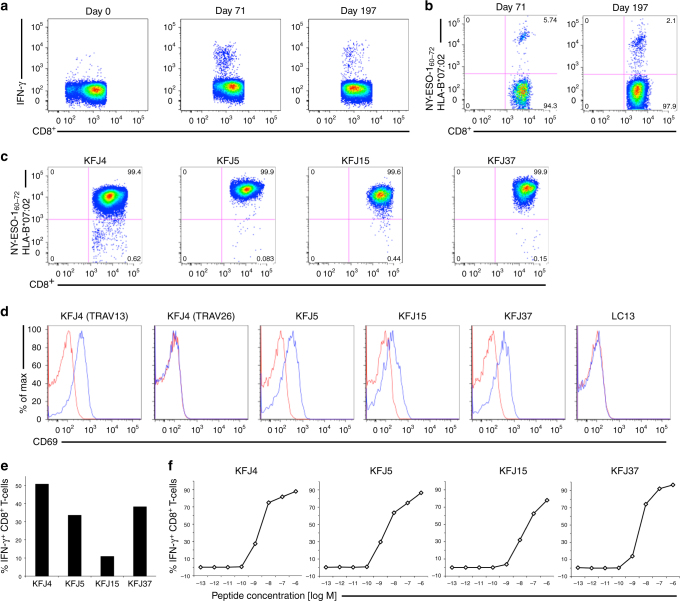
Functional reactivity of CD8^+^ T cells binding to HLA-B*07:02 presenting NY-ESO-1_60–72_. **a** Flow cytometry analysis of melanoma patient’s PBMCs showing CD8^+^ T-cell expansion pre- (Day 0) and post- (Days 71 and 197) NY-ESO-1-ISCOMATRIX^TM^ vaccination following in vitro stimulation with NY-ESO-1(55–72) peptide as assayed by intracellular cytokine staining (ICS) measuring interferon-γ (IFN-γ) production. **b** Flow cytometry analysis showing a distinct NY-ESO-1_60–72_-HLA-B*07:02 reactive CD8^+^ T-cell population following vaccination and in vitro stimulation with NY-ESO-1(55–72) peptide. Four functionally distinct NY-ESO-1_60–72_-HLA-B*07:02-specific CD8^+^ T-cell clones were identified from the melanoma patient’s PBMCs. **c** NY-ESO-1_60–72_-HLA-B*07:02 tetramer-positive CD8^+^ T-cell clones were generated from single-cell cloning. **d** Four NY-ESO-1_60–72_-HLA-B*07:02-specific TCRs were retrovirally reconstituted in SKW3 cells and recognised NY-ESO-1_60–72_-HLA-B*07:02 presented on peptide-pulsed HLA-B*07:02^+^ antigen-presenting cells (APCs). The overlaid flow cytometry histograms of CD69 up-regulation in TCR-transduced SKW3 cells following co-incubation with NY-ESO-1_60–72_ peptide-pulsed (blue histogram) or non-pulsed APCs (red histogram). The SKW3 subsets were gated on live lymphocytes, CD3^hi^ GFP^hi^ markers and were subsequently displayed as % of max (*Y*-axis) versus CD69 (*X*-axis). LC13 TCR-transduced SKW3 cells served as a TCR positive but nonreactive control as we have shown it recognises the EBNA-3A_339–347_ peptide-pulsed HLA-B*08:01^+^ APCs and up-regulated CD69 surface marker^44^. A second independent retroviral transduction experiment was performed for the KFJ4 (TRAV13), KFJ4 (TRAV26), KFJ37 and LC13 TCRs, with consistent results, see Supplementary Fig. 4. **e** Four different NY-ESO-1_60–72_-HLA-B*07:02-specific T-cell clones were individually co-incubated with HLA-B*07:02^+^ and NY-ESO-1-expressing SK-Mel-14 cells in 1:2 ratio in the presence of 10 μg mL^−1^ of Brefeldin A for 5 h to detect naturally presented NY-ESO-1_60–72_-HLA-B*07:02. CD8^+^ T-cell clone recognition of naturally presented NY-ESO-1_60–72_-HLA-B*07:02 on NY-ESO-1-expressing melanoma cell line, SK-Mel-14. CD8^+^ T-cell responses were assayed by ICS measuring IFN-γ production. **f** Functional sensitivity of the CD8^+^ T-cell clones to exogenous NY-ESO-1_60–72_ peptide at different concentrations. CD8^+^ T-cell clones that are sensitive to lower levels of antigen are considered to possess high avidity

To investigate the basis of extended peptide reactivity by polyclonal T cells from diverse TRBV families, we isolated and expanded NY-ESO-1_60–72_-HLA-B*07:02 reactive CD8^+^ T cells from peripheral blood mononuclear cells (PBMC) of patients with melanomas expressing NY-ESO-1. Following vaccination with NY-ESO-1 protein formulated with ISCOMATRIX^TM^ adjuvant^43^, an expansion of CD8^+^ T cells were observed at day 71, and persisted to day 197 post vaccination following in vitro stimulation with NY-ESO-1(55–72) peptide. Intracellular cytokine staining (ICS) measuring interferon-γ (IFN-γ) production was used to identify and enumerate the NY-ESO-1(55–72)-HLA-B*07:02 reactive cells within this expanded CD8^+^ T-cell pool (Fig. 1a). Previous peptide screening studies identified NY-ESO-1_60–72_ as the active 13-amino-acid epitope^17^. Thus, T cell specificity for NY-ESO-1_60–72_-HLA-B*07:02 in the expanded CD8^+^ T-cell pool was determined by tetramer staining. A similar trend in NY-ESO-1_60–72_-HLA-B*07:02 tetramer-positive T cells was seen post vaccination as for IFN-γ production within the T-cell pool (Fig. 1b). Further, CD3^+^ CD8^+^ NY-ESO-1_60–72_-HLA-B*07:02 tetramer-positive T cells were single cell sorted and expanded. TRBV gene usage of the T-cell clones was determined using a panel of Vβ specific antibodies (Supplementary Fig. 1a) and in-depth analysis of the paired α- and β-chain TCR gene usage of the sorted cells was done using TCR gene sequencing. To determine the paired α- and β-chain TCR gene usage of the isolated cells, TCR gene sequencing on four selected clones was performed (Table 1). Notably, no consensus motif/TCR bias was evident within the NY-ESO-1_60–72_-HLA-B*07:02-reactive TCR sequences in contrast to TCRs previously shown to recognise other extended peptides^12–16^.

**Table 1:** NY-ESO-1_60–72_-HLA-B*07:02-specific αβ TCR sequences

**CD8**^**+**^ **T-cell** clone	**TCR-α chain**					**TCR-β chain**						
	TRAV	TRAJ	CDR1α	CDR2α	CDR3α	TRBV	TRBD	TRBJ	TRBC	CDR1β	CDR2β	CDR3β
**KFJ4**	13-1*02	37*01	DSASNY	IRSNVGE	CAA***SL***NTGKLIF	10-3*02	1*01	1-2*01	1	ENHRY	SYGVKD	CAI***L***GQ***G***YGYTF
	26-1*01	50*01	TISGNEY	GLKNN	CIVR***GE***TSYDKVIF							
**KFJ5**	4*01	21*01	NIATNDY	GYKTK	CLVG***EILD***NFNKFYF	28*01	1*01	2-3*01	2	MDHEN	SYDVKM	CASS***QR***Q***EG***DTQYF
**KFJ15**	17*01	20*01	TSINN	IRSNERE	CA***TG***NDYKLSF	6-1*01	1*01	2-7*01	2	MNHNS	SASEGT	CAS***QTFSP***GQ***TQ***F
**KFJ37**	4*01	8*01	NIATNDY	GYKTK	CLV***VD***QKLVF	9*01	1*01	2-1*01	2	SGDLS	YYNGEE	CASS***G***G***HTGS***NEQFF

Non-germline encoded residues for the respective CDR3 regions are highlighted in bold italics

### Characterisation of NY-ESO-1_60–72_-HLA-B*07:02 reactivity

To confirm the NY-ESO-1_60–72_ reactivity of the HLA-B*07:02-restricted CD8^+^ T-cell clones, we selected four clones, all of which bound the NY-ESO-1_60–72_-HLA-B*07:02 tetramer (Fig. 1c). The clones represented a cross-section of the TCRα and TCRβ gene usage. These clones, KFJ4 (TRAV13-1^+^TRAJ37^+^ or TRAV26-1^+^TRAJ50^+^, TRBV10-3^+^ TRBD1^+^ TRBJ1-2^+^), KFJ5 (TRAV4^+^TRAJ21^+^, TRBV28^+^TRBD1^+^TRBJ2-3^+^), KFJ15 (TRAV17^+^TRAJ20^+^, TRBV6-1^+^ TRBD1^+^ TRBJ2-7^+^), and KFJ37 (TRAV4^+^TRAJ8^+^, TRBV9^+^TRBD1^+^ TRBJ2-1^+^), and suitable controls (the HLA-B*08:01-restricted TCR, LC13^44^) were used to generate a panel of TCR-transduced SKW3 T-cell lines. With the exception of the TRAV26-1^+^ KFJ4-SKW3 cells, these TCR-SKW3 cell lines up-regulated the T-cell activation marker CD69 upon stimulation with NY-ESO-1_60–72_ peptide-pulsed HLA-B*07:02^+^ antigen-presenting cells (APCs) (Fig. 1d). The KFJ4 T-cell clone carried two productive rearrangements of the TCR Vα-domain (Table 1) as identified in ~30% of T cells^45^, yet only the TRAV13-1^+^ TRAJ37^+^ α-chain (paired with TRBV10-3^+^ TRBD1^+^ TRBJ1-2^+^ Vβ-chain) formed a functional NY-ESO-1_60–72_-HLA-B*07:02 restricted TCR (Fig. 1d). The four functional CD8^+^ T-cell clones were confirmed to recognise naturally presented NY-ESO-1_60–72_-HLA-B*07:02 on the surface of SK-Mel-14 melanoma cells, as measured by induction of IFN-γ production (Fig. 1e). We then compared the functional reactivity of the TCR clones upon titrated NY-ESO-1_60–72_ peptide-pulsed APC stimulation. All four TCRs induced IFN-γ production at very low peptide concentrations (≤10^−9^ M) (Fig. 1f). Interestingly, the KFJ15 clone elicited a lower avidity response, consistent with the reduced level of staining (Fig. 1e) and faster dissociation (Supplementary Fig. 1b) of the NY-ESO-1_60–72_-HLA-B*07:02 tetramer.

**Fig. 2:**
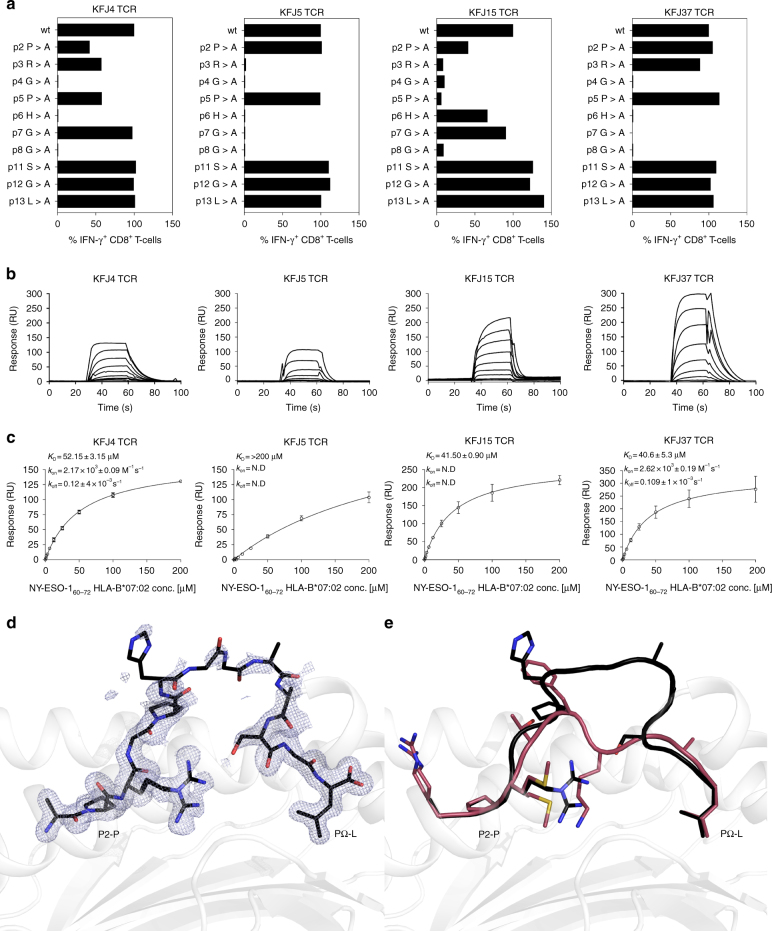
NY-ESO-1_60–72_ peptide specificities and steady-state affinities for CD8^+^ T-cell clones. **a** Normalised data for IFN-γ production by different CD8^+^ T-cell clones when stimulated with 10^−7^ M concentration of native NY-ESO-1_60–__72_ peptide (APRGPHGGAASGL) or single alanine-substituted peptides. CD8^+^ T cell responses stimulated by native peptide for each clones were accorded as 100%, and other CD8^+^ T cell responses stimulated by single alanine-substituted peptides were calculated as a relative percentage to the native peptide responses. CD8^+^ T cell responses stimulated by different single alanine-substituted peptides concentrations are shown in Supplementary Fig. 2. Surface plasmon resonance sensorgrams (**b**) and equilibrium binding curves (**c**) showed that the in vitro refolded and purified recombinant TCRs (immobilised at ~300–350 response units) interacted with NY-ESO-1_60__–72_-HLA-B*07:02 (screened at serial dilutions from 200 to 0 µM). SPR sensorgrams are representative of a single experiment. Equilibrium binding curves were derived from two replicate experiments with *K*_D_, *k*_on_, *k*_off_ and equilibrium curve means calculated from the duplicate experiments (error and error bars denote SEM). **d** The observed electron density (2mFo-DFc), shown as blue mesh, for the NY-ESO-1_60–72_ peptide contoured to 1*σ* illustrates the structural plasticity and flexibility of the central peptide residues (P6-His-P8-Gly). **e** NY-ESO-1_60–72_ adopts a bulged conformation upon presentation by HLA-B*07:02. Comparison of the NY-ESO-1_60–72_-HLA-B*07:02 bulged peptide presentation with the RL9-HLA-B*07:02 peptide presented in a canonical fashion (PDB: 5EO1). The heavy chain is shown in grey with the NY-ESO-1_60–72_ and RL9 peptides shown in black and red, respectively

To characterise the fine specificity of the identified T-cell clones specific to the NY-ESO-1_60–72_-HLA-B*07:02 complex on T-cell reactivity, we conducted an alanine-scanning assay. Here, the four T-cell clones displayed reduced reactivity to centrally mutated peptides (P4-Gly→P8-Gly), indicating the importance of the central region of the NY-ESO-1_60–72_ peptide (Fig. 2a and Supplementary Fig. 2) in TCR recognition^17^. Interestingly, the tolerance of KFJ5 and KFJ37 T-cell clones towards the P3-Arg mutation differed, with KFJ37 retaining ~90% of reactivity, whereas KFJ5 reactivity was completely ablated (Fig. 2a and Supplementary Fig. 2), suggesting that the KFJ5 and KFJ37 TCRs focused on the central region of the NY-ESO-1_60–72_ peptide presented by HLA-B*07:02 with differing fine specificities. We then examined the CD8 dependency of these TCRs, with each T-cell clone showing reduced activation when stimulated by NY-ESO-1_60–72_ peptide in the presence of a blocking CD8 antibody. This effect was most pronounced at low peptide concentrations (Supplementary Fig. 3) and served to highlight the importance of CD8 in NY-ESO-1_60–72_-HLA-B*07:02 restricted T-cell activation. CD8^+^ T-cell clones with higher avidity for NY-ESO-1_60–72_-HLA-B*07:02 tetramer (Supplementary Fig. 1) correlated well with their activation by lower peptide concentration (Fig. 1f) and by melanoma cells (Fig. 1e). Thus, fine specificity analysis indicated that the four TCRs exhibited different modes of interaction within the common central region of the NY-ESO-1_60–72_ epitope.

### Affinities for the NY-ESO-1_60–72_–HLA-B*07:02 complex

We next determined the affinity of the four TCRs towards the NY-ESO-1_60–72_–HLA-B*07:02 complex using surface plasmon resonance (SPR). Soluble ectodomains of the KFJ4, KFJ5, KFJ15 and KFJ37 TCRs, as well as the NY-ESO-1_60–72_–HLA-B*07:02 complex were expressed, refolded and purified^17,46^. As expected, the KFJ4, KFJ5, KFJ15 and KFJ37 TCRs did not bind to the control pHLA complex. The KFJ4, KFJ15 and KFJ37 TCRs bound to NY-ESO-1_60–72_-HLA-B*07:02 with an affinity (*K*_D_) of 40–50 μM (Fig. 2b, c). In contrast, the KFJ5 TCR bound with a markedly reduced affinity of >200 µM, suggesting greater structural challenges to TCR binding^14–16,20^. We next investigated the binding kinetics by determining the on-rate (*k*_on_), which were only measurable for the KFJ4 and KFJ37 TCRs. These values appeared notably slow when comparing the SPR sensorgrams and equilibrium binding curves (Fig. 2b, c) to other TCR-binding studies^14–16,20,47^. Similarly, the SPR sensorgrams for the KFJ5 and KFJ15 TCRs show a similarly pronounced slow association rate. The slow on-rates of these TCRs further suggest that TCR or peptide conformational rearrangements may be required for NY-ESO-1_60–72_ recognition, in contrast to TCR recognition of the rigid super-bulged LPEP peptide presented by HLA-B*35:08^16,20^.

### The NY-ESO-1_60–72_ peptide adopts a bulged conformation

To evaluate presentation of the NY-ESO-1_60–72_ peptide by HLA-B*07:02, we determined the structure of HLA-B*07:02 presenting the NY-ESO-1_60–72_ peptide to a resolution of 1.5 Å, which was significantly higher than the 2.1 Å resolution structure reported previously^17^ (Table 2). Superimposition of the HLA-B*07:02 molecule onto the structure of HLA-B*07:02 bound to a nonameric RFL9 peptide^48^ illustrated little movement in the HLA-I molecule (root-mean-squared deviation (r.m.s.d.) of 0.2 Å) (Fig. 2e), despite presenting distinct peptides. Given the anchored restraints at the peptide extremities, the central residues, encompassing P4-Gly→P11-Ser of the NY-ESO-1_60–72_ peptide, bulged extensively out of the peptide-binding groove. Here, the solvent accessible surface area of the NY-ESO-1_60–72_ peptide was double that of the RFL9 peptide (620 Å^2^ compared to 290 Å^2^), thereby providing an extended surface for TCR-mediated recognition. Notably, although solved to high resolution, P6-His-P8-Gly side chains were not clearly observed in the electron density (Fig. 2d), which suggested conformational plasticity in this region of the peptide.

**Table 2:** Data collection and refinement statistics

	**KFJ5 TCR**	**NY-ESO-1**_**60–72**_ **-HLA-B** ^*^ **07:02**	**KFJ5 TCR-NY-ESO-1**_**60–72**_ **-HLA-B** ^*^ **07:02**	**KFJ37 TCR-NY-ESO-1**_**60–72**_ **-HLA-B** ^*^ **07:02**
*Data collection*				
Temperature (K)	100	100	100	100
Space group	*P*2_1_ 2_1_ 2_1_	*P*2_1_ 2_1_ 2_1_	*P*2_1_	*P*1
*Cell dimensions*				
*a*, *b*, *c* (Å)	49.90, 73.36, 123.04	50.19, 82.07, 112.05	64.36, 67.69, 105.72	74.41, 66.66, 96.28
*α*, *β*, *γ* (°)	90, 90, 90	90, 90, 90	90, 102.93, 90	94.22, 97.88, 90
Resolution range (Å)	46.25–1.42 (1.49–1.42)	46.27–1.50 (1.58–1.50)	46.01–2.03 (2.14–2.03)	47.55–2.60 (2.74–2.60)
*R*_merge_	7.4 (48.7)	12.1 (56.4)	11.0 (57.9)	13.9 (68.3)
*I*/*σ*(*I*)	15.3 (3.8)	8.5 (2.9)	9.3 (2.5)	10.5 (2.2)
Completeness (%)	99.4 (97.8)	99.7 (100.0)	99.1 (96.8)	98.8 (98.2)
Redundancy	7.0 (6.8)	6.9 (7.2)	3.7 (3.7)	3.9 (3.9)
Total observations	603,854 (82,919)	517,968 (77,738)	211,802 (29,607)	217,862 (31,726)
Unique observations	86,034 (12,212)	74,669 (10,830)	57,083 (8094)	55,997 (8124)
*Refinement*				
*R*_work_^a^(%)	19.8	21.1	20.4	19.3
*R*_free_ (%)	21.8	23.0	25.6	24.8
*No. of atoms*				
Protein	3448	3191	6580	13,197
Water	618	912	447	561
*B-factors*				
Protein	27.2	19.6	40.8	39.5
Water	16.6	34.0	43.3	32.9
R.m.s. deviations				
Bond lengths (Å)	0.007	0.005	0.006	0.004
Bond angles (°)	1.096	0.952	1.171	1.050

Values in parentheses are for the highest-resolution shell

^a^ *R*_work_ = Σ_hkl_||*F*_o_| – |*F*_c_||/Σ_hkl_|*F*_o_| for all data excluding the 5% that comprised the *R*_free_ used for cross-validation

### Overview of TCR-NY-ESO-1_60–72_–HLA-B*07:02 complexes

**Fig. 3:**
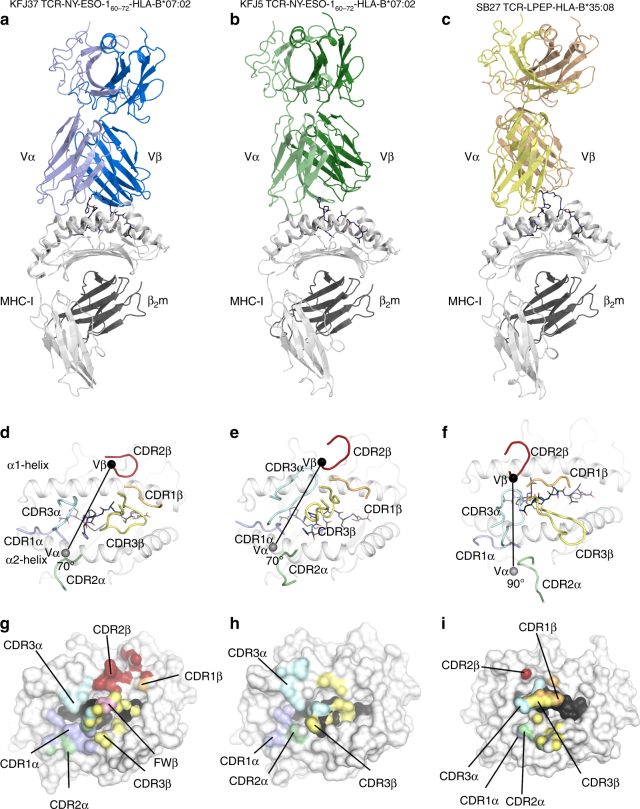
Overview of the ternary complexes. **a** KFJ37 TCR-NY-ESO-1_60–72_-HLA-B*07:02, **b** KFJ5 TCR-NY-ESO-1_60–72_-HLA-B*07:02 and **c** SB27 TCR-LPEP-HLA-B*35:08 (PDB: 2AK4) with the TCR α- and β-chains coloured lighter and darker shades of blue, green and yellow, respectively. The HLA-B heavy chain and β_2_-microglobulin molecules are shown as light and dark grey, respectively. Middle and lower panels show the TCR–HLA interfaces (**d**–**f**) and footprints (**g**–**i**). The complementarity determining region (CDR) loops are coloured as follows; CDR1α light blue, CDR2α green, CDR3α teal, CDR1β orange, CDR2β red, CDR3β yellow and framework (FW) contacts are coloured pink over the HLA-B*07:02 binding groove with the NY-ESO-1_60–72_ peptide shown as black sticks. The centre of mass of the respective Vα and Vβ domains are shown as grey and black spheres, respectively. In the lower panel the molecular surface of the HLA-B molecules is shown with atomic contacts coloured according to the respective CDR-loop mediated interactions

Next, we determined the structure of the KFJ5 and KFJ37 TCRs complexed to NY-ESO-1_60–72_-HLA-B*07:02 (Table 2). The well-defined electron density at the respective TCR-NY-ESO-1_60–72_–HLA-B*07:02 interfaces enabled the ternary complexes to be readily refined and interpreted. The KFJ37 TCR and KFJ5 TCR adopted similar and canonical docking modes, binding centrally atop the pHLA-I (Fig. 3a, b), with a docking angle of approximately 70° across the peptide-binding groove of HLA-B*07:02, whereby the Vα and Vβ of the TCR positioned over the α2- and α1-helices, respectively (Fig. 3d, e). In comparison to the KFJ37 TCR ternary complex, the KFJ5 TCR was shifted by ~2.5 Å towards the α1-helix, resulting in the Vα chain docking more centrally above the peptide-binding groove and the Vβ chain positioning more peripherally (Fig. 3e). The docking modes observed here differed notably from the SB27 TCR recognition of the LPEP–HLA-B*35:08 complex (Fig. 3c). Here, the SB27 TCR adopted a 90° docking angle and bound towards the N-terminus of the LPEP peptide and made limited contacts with the HLA-I molecule (Fig. 3f, i)^16^.

**Fig. 4:**
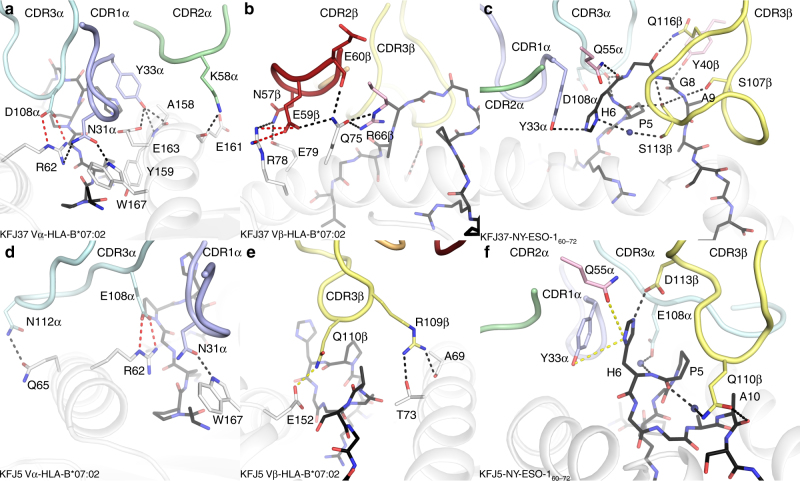
Molecular interactions at the TCR-NY-ESO-1_60–72_–HLA-B*07:02 interface. **a** KFJ37 α-chain and **b** β-chain interactions with HLA-B*07:02 and NY-ESO-1_60–72_ shown as grey cartoon and black sticks, respectively. **c** Interaction between the KFJ37 TCR and the NY-ESO-1_60–72_ peptide. Interaction between the KFJ5 α-chain (**d**) and β-chain (**e**) with HLA-B*07:02. **f** Interaction between the KFJ5 TCR and the NY-ESO-1_60–72_ peptide. The CDR loops are coloured according to Fig. 3. Hydrogen, van der Waals and salt-bridge contacts are shown by black, yellow and red dashed lines, respectively

The total buried surface area (BSA) of the KFJ37 TCR-NY-ESO-1_60–72_–HLA-B*07:02 interface was 2350 Å^2^, of which the TCR α- and β-chains contributed approximately equally (45% and 55%, respectively) (Fig. 3g). The KFJ37 TCR NY-ESO-1_60–72_–HLA-B*07:02 interface comprised residues from both germline and non-germline residues with each CDR loop of the α-chain (20%, 7%, 15% and 1% BSA from the CDR1α, CDR2α, CDR3α and FW, respectively) and β-chain (2%, 18%, 26% and 9% BSA from the CDR1β, CDR2β, CDR3β and FW, respectively) contributing to HLA-B*07:02 recognition (Fig. 3g). Here, Asn31α and Tyr33α (CDR1α) projected down towards the HLA-I surface and formed hydrogen bonds to Arg62 and Trp167, and Ala158, Tyr159 and Glu163 of the HLA-I, respectively (Fig. 4a and Supplementary Table 1). The CDR1α contacts to the HLA-I surface were further stabilised via extensive van der Waals contacts involving Asn31α and Tyr33α, in addition to Thr30α contacting Glu163, Glu166, Trp167 of the HLA-I α2-helix. Further, Lys58α (CDR2α) formed a hydrogen bond to Glu161 of the HLA-I α2-helix. Additionally, Asp108α (CDR3α) formed a salt bridge with Arg62 of the α1-helix (Fig. 4a and Supplementary Table 1). The β-chain of the KFJ37 TCR makes further ionic interactions with the HLA-I α1-helix, whereby Glu59β (CDR2β) salt bridged to the protruding Arg78 (Fig. 4b and Supplementary Table 1). This interaction surface was further stabilised via hydrogen bonds from Glu60β (CDR2β) and Arg66β (FWβ) to the neighbouring residue on the α1-helix, Gln75 (Fig. 4b and Supplementary Table 1).

**Fig. 5:**
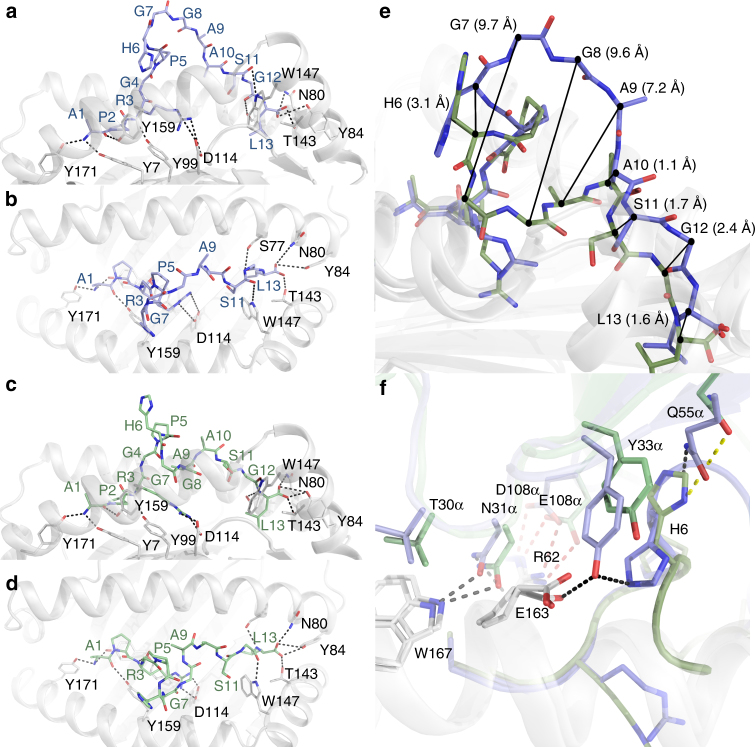
Conformational plasticity of the NY-ESO-1_60–72_ peptide dictates TCR recognition modes. **a** Side view of the NY-ESO-1_60–72_ peptide as presented for KFJ37 TCR-mediated recognition and **b** top view. **c** Side view of the NY-ESO-1_60–72_ peptide as presented for KFJ5 TCR-mediated recognition and **d** top view. HLA-B*07:02 shown as grey cartoon with the NY-ESO-1_60–72_ peptide shown as sticks coloured, blue and green for the KFJ37 TCR and KFJ5 TCR recognised epitopes. **e** Overlay of the two NY-ESO-1_60–72_ peptide conformers with the carbon-a deviations shown for the entire peptide (**f**). The conserved interaction codon of the TRAV4*01 variable domain with the KFJ37 TCR and KFJ5 TCR coloured blue and green, respectively. Hydrogen and van der Waals bonds are shown by black and yellow lines with the conserved non-germline encoded Asp/Glu108α salt bridge also shown, in red

The total BSA of the KFJ5 TCR-NY-ESO-1_60–72_–HLA-B*07:02 interface was notably smaller at 1560 Å^2^. Here, the KFJ5 TCR ternary interface was dominated by TCR α-chain contacts, which contributed ~75% of the BSA. The CDR1α, CDR2α, CDR3α and FWα loops contributed 30%, 4%, 38% and 2%, respectively, to the BSA of the interface, whereas only the CDR3β of the β-chain contributed (25% BSA) to the interface (Fig. 3h and Supplementary Table 2). The KFJ5 β-chain formed limited contacts with the HLA-I surface and comprised contacts only from the CDR3β loop, including Arg109β, which contacted Thr73 and the main chain of Ala69 of the α1-helix, and Gln110β, which contacted Glu152 of the α2-helix (Fig. 4e and Supplementary Table 2). In the KFJ5 TCR ternary complex, contact to the HLA-I surface was facilitated via an extension of the F-strand of the KFJ5 Vα domain over the N-terminal extremity of the α1-helix (Fig. 4d). Here the CDR3α loop is distended through the incorporation of four additional amino acids whereby the non-germline residues served to cap the peptide-binding groove of the HLA-I along with the germline Asn112α (CDR3α), which formed a hydrogen bond with Gln65 of the α1-helix (Fig. 4d and Supplementary Table 2). Given the commonality of the TRAV4*01 chains between the KFJ37 and KFJ5 TCRs, it was of interest that there were some conserved TRAV4*01-mediated contacts. Namely, Thr30α of the CDR1α made conserved van der Waals contacts to Gln163, Glu166 and Trp167 (Fig. 5f). Further, the neighbouring Asn31α made a conserved hydrogen bond to Trp167 and contacts with Arg62 (Fig. 5f). Interestingly, Tyr33α made conserved van der Waals contact to Glu163, as well as conserved coordination of P6-His (Fig. 4c, f), In addition, the TRAV4*01-encoded Thr30α and Gln55α made conserved contacts to the P6-His of the peptide (Fig. 5f). Further, non-germline commonalities included a salt-bridge contact from Asp/Glu108α to Arg62 of the HLA-B*07:02 α1-helix (Fig. 5f). Thus, while the KFJ37 and KFJ5 TCRs share the same TRAV-encoded region and adopt a similar positioning atop the pHLA-I, the extent and details within the respective interfaces differ markedly, despite recognising the same HLA–peptide complex.

### Differential TCR recognition of the NY-ESO-1_60–72_ peptide

The position of the KFJ37 TCR CDR loops on the HLA-B*07:02 surface allows the TCR to completely encapsulate the protruding NY-ESO-1_60–72_ peptide (Fig. 4c). Indeed, the central part of the peptide was sandwiched between both CDR3 loops and three-tyrosine residues from CDR1α, FWβ and CDR2β (Supplementary Table 1). As observed in the binary structure, the central portion of the NY-ESO-1_60–72_ peptide arched out of the peptide-binding groove, yet here was stabilised upon KFJ37 TCR ligation (Fig. 4c). Namely, the central region (P6-His→P9-Ala) of the NY-ESO-1_60–72_ peptide formed extensive contacts with the KFJ37 TCR. Here, P6-His made hydrogen bonding contacts with Tyr33α of the CDR1α loop and Gln55α of the FWα (Fig. 4c and Supplementary Table 1) and also made a water-mediated contact to Ser113β of the CDR3β. The bulging peptide was further stabilised by Ser107β and Ser113β of the CDR3β loop, which formed hydrogen bonds to the peptide main chain at positions P9-Ala, and P8-Gly, respectively, in addition to numerous van der Waals contacts to the peptide (Fig. 4c and Supplementary Table 1). Indeed, the bulging peptide comprised 36% of the interaction surface (BSA) for KFJ37 TCR-mediated HLA-B*07:02 recognition, comparable to the ~41% contribution to peptide-mediated recognition in SB27 TCR^12^.

In stark contrast, the NY-ESO-1_60–72_ peptide underwent dramatic remodelling upon recognition by the KFJ5 TCR, due in part to the steric occlusion from the distended CDR3α loop and the coordination of the CDR3β loop. Here, the super-bulged NY-ESO-1_60–72_ topology was deformed upon KFJ5 TCR ligation and adopted a ‘helical’ peptide conformation (P5-P8) (Fig. 4f), the impact of which resulted in a relatively flattened epitope upon KFJ5 TCR engagement, which in turn resulted in a markedly reduced (~20%) contribution to the interaction surface. Peptide recognition in the KFJ5 TCR ternary complex was almost entirely modulated via van der Waals interactions, notwithstanding the solitary hydrogen bond to the peptide from Asp113β of the CDR3β to P6-His, which flipped 180° into an upright conformation, compared to the KFJ37 TCR complex, while retaining contacts with Tyr33α of the CDR1α (Fig. 4f and Supplementary Table 1). The large deformation of the bulged peptide is coordinated via water-mediated interactions from the KFJ5 TCR CDR3 loops with Glu108α and Gln110β serving to stabilise the helical peptide main chain at P6-His and P5-Pro, respectively (Fig. 4f and Supplementary Table 2). Van der Waals interactions included Glu108α, Ile109α and Leu110α of the CDR3α, to P4-Gly and P5-Pro of the peptide, which further contributed to the peptide interface with the α-chain of the KFJ5 TCR (Fig. 4f and Supplementary Table 2). Conversely, the β-chain made limited contribution to the TCR–peptide interface, with only Arg109β and Gln110β of the CDR3β loop making van der Waals contacts with P10-Ala and P11-Ser peptide side chains (Supplementary Table 2).

Superimposition of the two-peptide conformations revealed an r.m.s.d. value of 3.6 Å over the 81 atoms of the peptide, representing a considerable remodelling (Fig. 5a–d). While the N-terminus of the NY-ESO-1_60–72_ peptides remained unchanged from P1-Ala→P5-Pro (Fig. 5a–d), the coordination chemistry of the peptide backbone shifted drastically through positions P6-His, P7-Gly, P8-Gly and P9-Ala with the α-carbon of each residue moving 3.1, 9.7, 9.6 and 7.2 Å, respectively (Fig. 5e). Taken together, these structures indicated that the NY-ESO-1_60–72_ peptide was highly malleable prior to engagement by two cognate TCRs.

### Peptide plasticity is complemented by TCR malleability

To further understand the remodelling at the TCR-NY-ESO-1_60–72_–HLA-B*07:02 interface, we determined the structure of the KFJ5 TCR in an unligated form (Table 2). When comparing the KFJ5 TCR in a ligated and unligated form (Supplementary Fig. 5a), the CDR regions underwent significant remodelling. A large movement of the CDR3α was observed in which residues Glu108α to Phe116α (Supplementary Fig. 5b & d) moved ~9 Å upon recognition of the NY-ESO-1_60–72_–HLA-B*07:02 complex. A more modest remodelling was observed within the CDR regions of the β-chain, whereby the CDR3β displayed the largest shift of ~2 Å upon ligation (Supplementary Fig. 5c & d). The pronounced movement of the KFJ5 TCR CDR3α loop is indeed prerequisite for HLA recognition, due to major steric clashes with P5-Pro and P6-His of the NY-ESO-1_60–72_ peptide. The remodelling suggests that the structural plasticity of extended epitopes can be accommodated to some extent by complementary remodelling of the TCR, but at an energetic cost, as illustrated by the reduced *K*_D_ of the KFJ5 TCR.

## Discussion

A generic tenet of T-cell-mediated immunity is the ability of the TCR to mould around a given HLA-I molecule when presenting a peptide of canonical length^1,46,49^. However, our understanding of TCR recognition towards extended HLA-I restricted peptides is unclear despite their growing importance in T-cell-mediated immunity^6^. Two contrasting TCR recognition modes have been described for such extended peptides. Firstly, a TCR was observed to perch atop a rigid and super-bulged 13-amino-acid peptide, thereby making limited contacts with the HLA-I molecule^12,16^. This same epitope was recognised differently by a distinct TCR, but the epitope retained its rigid conformation^16^. Secondly, a TCR flattened a bulged peptide upon recognition to enable a more conventional HLA-I centric footprint^13^. We describe a polyclonal CD8^+^ T cell response to HLA-B*07:02 presenting the immunodominant NY-ESO-1_60–72_ peptide, which was detected in patients with melanomas expressing NY-ESO-1^17^, an antigen of importance in onco-immunology given its tumour-dependent expression and growing importance as a therapeutic target^39,43,50,51^.

The structural basis for which distinct TCRs can recognise the same pHLA-I complex has been described^12,16,51–55^. Two TCRs (A6 and B7) bound Tax-HLA-A*02:01 in a largely conserved docking mode despite differing TRAV gene usage^52^. Similarly three TCRs (H27-14, T36-5 and C1-28)^53^ and two TCRs (DMF4 and DMF5)^51^, which utilised varied TRAV and TRBV gene segments, recognised RFP-HLA-A*24:02 and MART-1-HLA-A*02:01, respectively, with a conserved docking mode^51,53^. In both cases the variation at the TCR–pHLA-I interface contributed to altered interatomic contacts thereby providing a molecular basis for differing TCR functional responses to pHLA-I stimulation. In contrast, varied TCR gene usage directed against FLR-HLA-B*08:01 resulted in differing docking topologies^44,54,55^. Here, the RL42 TCR employed a central docking mode, whereas the CF34 and LC13 TCRs docked towards the N-terminus and C-terminus of the peptide, respectively^44,54,55^. Similar observations have been observed for TCR recognition of HLA-B*35:08 presenting an extended 13-amino-acid LPEP^12,16^.

Here we demonstrate a new mechanism of TCR recognition of the same pMHC-I complex. Namely, we show how a polyclonal TCR repertoire directed against the same pHLA-I complex can either maintain or deform the conformation of the extended epitope. The KFJ37-TCR bound NY-ESO-1_60–72_-HLA-B*07:02 docking centrally atop the bulged central region of the peptide, stabilising the dynamic central portion of the NY-ESO-1_60–72_ peptide between the Vα and Vβ domains of the KFJ37-TCR. Conversely the KFJ5-TCR adopted an alternate recognition mode, whereby the bulged peptide was flattened upon TCR ligation. Although the identified NY-ESO-1_60–72_-HLA-B*07:02 restricted T-cell population was characterised by a diverse TCR gene repertoire, the KFJ37 and KFJ5-TCRs utilised the same TRAV4*01 gene. In spite of differing peptide conformers being recognised by alternate peptide docking modes there were some vestiges of conserved pairwise^56,57^ germline contacts between the TRAV4*01-encoded TCR α-chain and the HLA-B*07:02 molecule and the peptide itself.

Our ternary structures highlighted the extent to which TCR plasticity can be harnessed by the adaptive immune system to evoke an immune response. Our findings provide novel insight into the recognition of extended peptide epitopes via distinct T-cell populations, and highlight their utilisation of widely differing docking modes to recognise an oncogenic epitope of significant clinical interest.

## Methods

### NY-ESO-1_60–72_-HLA-B*07:02-specific CD8^+^ T-cell isolation

Patient peripheral blood cells were obtained with informed consent and the study was approved by the Human Research Ethics Committee of Austin Health (ethics approval number: H2001-01256 and title: Immunological characterisation of patients with cancer)^43^. PBMCs were isolated by density gradient centrifugation (Ficoll-Pague Plus, GE Healthcare). Approximately 3 × 10^6^ PBMCs from days 71, 197 and 547 post-NY-ESO-1/ISCOMATRIX vaccination trial samples were pulsed with NY-ESO-1_55–72_ peptide GPGGGAPRGPHGGAASGL (10 μg mL^−1^), for 1 h at 37 °C. The cells were then cultured in a 24-well plate in 2 mL of RPMI-1640 (Gibco) containing 10% (v/v) FCS (Thermo Trace), recombinant human IL-2 (25 U mL^−1^, Proleukin), Glutamax (2 mM), benzylpenicillin (100 U mL^−1^), streptomycin (100 µg mL^−1^), HEPES buffer (10 mM), 2-mercaptoethanol (50 µM), sodium pyruvate (1 mM) and 1 × nonessential amino acids (all from Gibco), harvested on days 12–15 for NY-ESO-1_60–72_-HLA-B*07:02 tetramer staining and ICS. ICS assay was performed by stimulating CD8^+^ T cells with diluted synthetic peptide in 200 μL of complete RPMI-1640 medium (described above) in 10 μg mL^−1^ Brefeldin A (BFA; Sigma-Aldrich) and incubated for 5 h at 37 °C. Cells were stained with 50 μL of PBS-diluted (1:50) antihuman CD3 and CD8 (BD Biosciences) for 30 min at 4 °C. The monoclonal antibody target, clone name and source are presented in Supplementary Table 3. Cells were washed once with 100 μL PBS and were resuspended in 100 μL 1% paraformaldehyde (PFA; EMS) for 20 min at room temperature. Cells were washed once with PBS and stained with 50 μL of PBS-diluted (1:100) antihuman IFN-γ (BD Biosciences) in 0.2% saponin (Sigma-Aldrich) for 30 min at 4 °C. Cells were washed once with PBS, resuspended in 100 μL PBS and analysed using FACSCanto II flow cytometer (BD Biosciences). Data were processed with FlowJo (Tree Star Inc.) software. NY-ESO-1_60–72_-HLA-B*07:02 tetramer staining was performed by staining cells with 100 μL of PBS-diluted (1:150) NY-ESO-1_60–72_-HLA-B*07:02 tetramer (0.44 mg mL^−1^) for 30 min at room temperature. Tetramers were synthesised at the Tetramer Production Facility of the Ludwig Institute for Cancer Research (LICR). Tetramer-stained cells (~2 × 10^6^) were further stained with antibodies specific to CD3 and CD8 (BD Biosciences). Tetramer and antibodies were used at an empirically determined dilution factor with an optimum signal to noise ratio. Tetramer^hi^, CD3^hi^ and CD8^hi^ cells were individually sorted using FACSAria III flow cytometer (BD Biosciences) into a round-bottom 96-well plate containing 200 µL of complete RPMI-1640 medium (as described above but without FCS) supplemented with 10% (v/v) heat-inactivated human AB sera (LICR), 600 U recombinant human IL-2, phytohaemagglutinin (1 μg mL^−1^, Sigma-Aldrich) and 3 × 10^5^ healthy donors’ PBMCs (Australian Red Cross Blood Service). PBMCs were mixed from three different donors in 1:1:1 ratio before being irradiated at 3000 rad using Gammacell 1000 Elite Irradiator ^137^Cs source (Nordion). The gating strategy used to sort these cells is presented in Supplementary Fig. 6a. Tetramer^+^ CD8^+^ clonal cells were further expanded on day 15 post cell-sorting for another 10 to 12 days in the same setting as described above, with ~5000 clonal cells transferred into each well on a round-bottom 96-well plate initially.

### Characterising the CD8^+^ T-cell clone TCRβ repertoire

All NY-ESO-1_60–72_-HLA-B*07:02-specific CD8^+^ T-cell clones were stained with a panel of 24 antibodies specific to TCR Vβ chains (Beckman Coulter) and analysed using FACSCanto II flow cytometer (BD Biosciences), and data were processed with FlowJo (Tree Star Inc.) software. The gating strategy used to analyse these cells is presented in Supplementary Fig. 6b.

### T cells recognise NY-ESO-1_60–72_-HLA-B*07:02 on melanoma cells

SK-Mel-14, an HLA-B*07:02^+^ melanoma line that expresses NY-ESO-1 antigen^17^ (Memorial Sloan-Kettering Cancer Centre, USA), and LM-Mel-26, an HLA-B*07:02^+^ melanoma line that does not express NY-ESO-1 antigen^17^ (LICR), were treated with recombinant human IFN-γ (50 ng mL^−1^, R&D Systems) for 48 h. CD8^+^ T-cell clones were co-incubated with these melanoma lines in 1:2 ratio in 10 μg mL^−1^ of Brefeldin A (BFA, Sigma-Aldrich) for 5 h followed by antibody staining for CD3, CD4 and CD8 (BD Biosciences), and ICS. The gating strategy used to analyse these cells is presented in Supplementary Fig. 6c. All cell lines were tested and/or treated for mycoplasma and were mycoplasma free.

### Peptide titration and alanine-scanning analyses

Serial log dilutions of NY-ESO-1_60–72_ and single alanine-substituted peptides at 1 μM down to 10^−13^ M in RPMI-1640 medium were prepared. All peptides were synthesised with purity >95% (Mimotopes, Australia). CD8^+^ T-cell clones were co-incubated with LM-Mel-26 (as APCs) in 1:2 ratio in serially diluted peptide and BFA (10 μg mL^−1^) for 5 h followed by antibody staining and ICS (described above). The gating strategy used to analyse these cells is presented in Supplementary Fig. 6c.

### Tetramer dissociation assay

CD8^+^ T-cell clones (~10^5^) were stained with NY-ESO-1_60–72_-HLA-B*07:02 tetramer for an hour. Cells were then washed three times with ‘cold buffer’ [PBS (Gibco) containing 0.5% (w/v) bovine serum albumin (Gibco) and EDTA (2 mM, Sigma-Aldrich)] before being resuspended in RPMI-1640 with 10% (v/v) FCS medium containing unconjugated anti-HLA-B7 (Serotec), and were incubated at 37 ^o^C. At designated time points (0, 5, 15, 30, 45, 60, 90, 120, 150, 180, 240 and 300 min), cells were harvested, washed for three times with cold buffer and stained with anti-CD8 (BD Biosciences) before flow cytometric analysis. The gating strategy used to analyse these cells is presented in Supplementary Fig. 6e.

### CD8 co-receptor dependence assay

CD8 co-receptors on CD8^+^ T-cell clones were blocked with unconjugated anti-CD8 (Serotec) for an hour. The treated CD8^+^ T-cell clones were incubated with or without P03-BLCL, an HLA-B*07:02^+^ Epstein-Barr virus transformed B-cell lymphoblastoid line (LICR) in 1:2 ratio in serially diluted NY-ESO-1_60–72_ peptide and BFA (10 μg mL^−1^) for 5 h, followed by antibody staining and ICS (described above). Two other closely related assay conditions were set up for the same CD8^+^ T-cell clones without anti-CD8 blocking. The gating strategy used to analyse these cells is presented in Supplementary Fig. 6c.

### TCR clonotyping and αβ TCR cell line generation

RNA was prepared from CD8^+^ T-cell clones with Trizol (Invitrogen) and was reverse transcribed with 5′ rapid amplification of cDNA end (RACE, Invitrogen) using gene-specific primers, Cα-GSP1 (GGGAAGAAGGTGTCTTCTGGAAT) and Cβ-GSP1 (GGCTGCTCAGGGCGTA). DNA fragments containing the sequence encoding the CD8^+^ T-cell clone’s α- or β-chains were obtained by 5′ RACE PCR amplification of cDNA with combinations of the 5′ RACE abridged anchor (Invitrogen) and gene-specific primers, Cα-GSP2 (GCTGTTGTTGAAGGCGTTTGC) or Cβ-GSP2 (GTGGCCAGGCATACCAGTGT). Each PCR-derived α or β gene was cloned into pGEM-T Easy vector (Promega) and sequenced. Five full-length genes encoding TCR α- and β-chains (Table 1) separated by a hydrolase element P2A linker^58^ were synthesised (GenScript) and subcloned into the pMIG expression vector^58^. SKW3 cells, a TCR α/β^−/−^ and derivative of KE-37 cells of acute lymphoblastic leukaemia (Leibniz Institute DSMZ-German Collection of Microorganisms and Cell Cultures) were transduced with vectors encoding the sequenced αβ TCRs (Table 1, Supplementary Fig. 4) or the control LC13 TCR^44^. Retrovirus transduction was performed by first plating ~10^6^ HEK 293 T cells in tissue culture plate in 10 mL of complete DMEM medium containing 10% (v/v) FCS (Thermo Trace), Glutamax (2 mM), benzylpenicillin (100 U mL^−1^), streptomycin (100 µg mL^−1^), HEPES buffer (10 mM), 2-mercaptoethanol (50 µM), sodium pyruvate (1 mM) and 1 × nonessential amino acids (all from Gibco), and were incubated overnight at 37 °C. 4 µg TCR 2A-linked/pMIG, 2 µg pVSV-G^58^ and 4 µg pPAME^58^ plasmids were dropwise added into a microtube containing 470 μL OptiMEM medium (Invitrogen) and 30 μL Fugene 6 reagent (Roche), gently tapped to mix and incubated at room temperature for 15 min. The mixture was dropwise added to overnight-cultured HEK 293 T cells for plasmid co-transfection and further incubated overnight at 37 °C. Cell culture medium of the overnight transfected HEK 293 T cells was replaced with 10 mL of complete DMEM medium. About 10^5^ SKW3 cells were seeded in tissue culture flask in 10 mL of complete DMEM medium and incubated overnight at 37 °C. After 12 h incubation, HEK 293 T-cell culture medium containing retroviruses was carefully harvested and filtered through a 0.45 μm filter (Pall) to resuspend SKW3 cells and 10 μL polybrene (6 mg mL^−1^, Sigma-Aldrich) was added to the resuspended cells. Ten millilitres of complete DMEM medium was added to the HEK 293 T cells and incubated at 37 °C. This process was repeated for another six times in every 12 h interval. Finally, TCR-transduced SKW3 cell culture medium was carefully aspirated, cells were washed three times with PBS, resuspended in complete DMEM and further incubated at 37 °C for 7–10 days. For T-cell activation assays, TCR-transduced SKW3 cells (~10^5^) were co-incubated with peptide-pulsed P03-BLCL cells (pre-pulsed with 10^−5^ M NY-ESO-1_60–72_ peptide for 2.5 h at 37 °C) in 1:1 ratio for 5 h at 37 °C. P03-BLCL cells without peptide pulsing were kept as a control. Cells were then stained with antibodies specific to CD3 and CD69 (BD Biosciences), washed once with PBS and subsequently fixed in 1% (v/v) paraformaldehyde (EMS) before flow cytometric analysis. The gating strategy used to analyse these cells is presented in Supplementary Fig. 6d.

### Protein expression and purification

Gene sequences encoding the functional NY-ESO-1_60–72_-HLA-B*07:02-specific TCR α- and β-chains were engineered with the avidex disulphide linkage in the constant region (KFJ4 TCR: Thr159αCys, Ser168βCys, Cys186βAla; KFJ5 TCR: Thr160αCys, Ser170βCys, Cys188βAla; KFJ15 TCR: Thr157αCys, Ser169βCys, Cys187βAla; KFJ37 TCR: Thr155αCys, Ser171βCys, Cys189βAla), codon-optimised (GenScript) and subcloned into the pET-30 expression vector (Novagen). The KFJ4, KFJ5, KFJ15 and KFJ37 TCR heterodimers were expressed as inclusion bodies in BL21 *Escherichia coli* and solubilised in 8 M urea, 20 mM Tris–HCl pH 8, 0.5 mM EDTA and 1 mM DTT. TCR heterodimers were refolded by flash dilution into 5 M urea, 100 mM Tris–HCl pH 8.0, 2 mM EDTA, 400 mM l-arginine-HCl, 0.5 mM oxidised glutathione and 5 mM reduced glutathione before dialysis into 10 mM Tris–HCl pH 8.0 and purified by gel filtration and anion-exchange chromatography^46^. Soluble HLA-B*07:02 heterodimers complexed to NY-ESO-1_60–72_ were produced by expressing a truncated form of the HLA-B*07:02 heavy chain and human β2m as inclusion bodies and purified after oxidative refolding by gel filtration and anion-exchange chromatography^17^. Conformational integrity of the purified TCRs and NY-ESO-1_60–72_-HLA-B*07:02 proteins was assessed using the conformationally sensitive monoclonal antibodies 12H8 and W6/32, respectively^46^.

### SPR

SPR experiments were conducted at 20 °C on a BIAcore 3000 instrument in 10 mM HEPES, pH 7.4, 150 mM NaCl supplemented with 0.005% (v/v) surfactant P20 and 1% BSA. The analyte was captured on a CM5 sensorchip using the conformational monoclonal antibody 12H8^59^, whereby amine-coupled 12H8 was used to immobilise the KFJ4, KFJ5, KFJ15 and KFJ37 TCRs to a surface density of ~300–350 response units. Serial dilutions from 200 to 0 µM of NY-ESO-1_60–72_-HLA-B*07:02 were injected over the immobilised TCR. The antibody surface was regenerated with Actisep (Sterogene) between two injections. All experiments were performed in duplicate with the kinetic parameters calculated using the BIAevaluation program using 1:1 Langmuir binding models with the addition of a drifting baseline parameter.

### Crystallisation and structure determination

All crystals were grown by the hanging-drop, vapour-diffusion method at 20 °C, crystals were flash-frozen in mother liquor supplemented with 30% (w/v) of the respective PEG, and data were collected at the Australian Synchrotron MX1 and MX2 beamlines, Melbourne. Data were processed with the program XDS^60^ and were scaled with the SCALA program of the CCP4 suite^61^. Orthorhombic crystals of the KFJ5 TCR were grown in 0.1 M Tris–HCl pH 8.5 and 25% (w/v) PEG 3350 in 1:1 mixture with 8 mg mL^−1^ protein. KFJ5 TCR crystals belonged to the *P*2_1_2_1_2_1_ space group and diffracted to 1.4 Å. Molecular replacement was conducted with the TRBV28^+^ TCR HA1.7 (PDB code 1J8H)^62^ as the search ensemble. An initial run of rigid body refinement was performed using the Phenix REFINE software^63^, the CDR loops of the TCR were built in COOT^64^. Iterative rounds of refinement cycles lead to a final model with an *R*_work_ and *R*_free_ of 19.8% and 21.8%, respectively. Orthorhombic crystals of the NY-ESO-1_60–72_-HLA-B*07:02 complex were grown in 0.2 M NaCl and 20% (w/v) PEG 3350 at a 1:1 mixture with 5 mg mL^−1^ protein. The NY-ESO-1_60–72_-HLA-B*07:02 crystals belonged to the *P*2_1_2_1_2_1_ space group and diffracted to 1.5 Å. Molecular replacement was conducted with RFL9-HLA-B*07:02 (PDB code 5eo0)^48^ as the search ensemble. An initial run of rigid body refinement was performed using Phenix REFINE^63^ to find optimal placement of the HLA-B*07:02 and β2m. Iterative rounds of refinement cycles lead to a final model with an *R*_work_ and *R*_free_ of 21.1% and 23.0%, respectively. Triclinic crystals of the KFJ37 TCR-NY-ESO-1_60–72_-HLA-B*07:02 and monoclinic crystals of the KFJ5 TCR-NY-ESO-1_60–72_–HLA-B*07:02 complexes were grown in 0.1 M MIB pH 7 and 25% (w/v) PEG 1500, and 0.1 M BTP pH 8.8, 0.2 M Na-Iodide, 14% (w/v) PEG 3350 and 2% (w/v) PEG 20,000, respectively, at a 1:1 mixture with 10 mg mL^−1^ protein. The KFJ37 TCR-NY-ESO-1_60–72_-HLA-B*07:02 and KFJ5 TCR-NY-ESO-1_60–72_–HLA-B*07:02 complexes belonged to the *P*1 and *P*2_1_ spacegroups, and diffracted to 2.6 and 2.0 Å, respectively. Molecular replacement was conducted with the refined structures of the KFJ5 TCR and NY-ESO-1_60–72_-HLA-B*07:02 serving as separate search ensembles. The electron density at the interface was clear and unambiguous. Initial rounds of rigid body refinement were performed using Phenix REFINE^63^. The CDR loops of the respective TCRs were built in COOT^64^, where iterative rounds of refinement cycles lead to final models with an *R*_work_ and *R*_free_ of 19.3 and 24.3% for the KFJ37 TCR-NY-ESO-1_60–72_–HLA-B*07:02 complex and 20.4 and 25.6% for the KFJ5 TCR-NY-ESO-1_60–72_–HLA-B*07:02 complex. Atomic contacts were determined with the CONTACT program from the CCP4i suite^61^. The quality of all structures was confirmed at the Research Collaboratory for Structural Bioinformatics Protein Data Bank Data Validation and Deposition Services website. All presentations of molecular graphics were created with the PyMOL molecular visualisation system (The PyMOL Molecular Graphics System, Version 1.5.0.4 Schrödinger, LLC.

### Data availability

The refined coordinate and structure factors files for the X-ray crystal structures of the NY-ESO-1_60–72_-HLA-B*07:02, KFJ5 TCR, KFJ37 TCR-NY-ESO-1_60–72_-HLA-B*07:02 and KFJ5 TCR-NY-ESO-1_60–72_-HLA-B*07:02 structures have been validated using the Protein Data Base validation site and the coordinates relating to the data reported in this study were deposited in the protein data bank with identification codes 6AT5, 6AT6, 6AVG, 6AVF, respectively. All remaining data are available within the article and its Supplementary Information files and from the corresponding authors on reasonable request.

## Electronic supplementary material

Supplementary Information
