# Supplementary material for: Divergent T-cell receptor recognition modes of a HLA-I restricted extended tumour-associated peptide
Source: Nat Commun. 2018 Mar 12;9:1026. doi: 10.1038/s41467-018-03321-w (PMC5847591; doi:10.1038/s41467-018-03321-w)
Supplement: Supplementary file 1 — Supplementary Information [file 41467_2018_3321_MOESM1_ESM.pdf]

Divergent T cell receptor recognition modes of a HLA-I restricted extended tumour-associated peptide

Kok Fei Chan[1,2,3,4*], Benjamin S. Gully[3,5*], Stephanie Gras[3,5*], Dennis X. Beringer[3], Lars Kjer-Nielsen[2], Jonathan Cebon[1], James McCluskey[2], Weisan Chen[1,4#] & Jamie Rossjohn[3,5,6#]

[1]Olivia Newton-John Cancer Research Institute, and School of Cancer Medicine, La Trobe University, Heidelberg. Victoria 3084, Australia

[2]Department of Microbiology and Immunology, Peter Doherty Institute for Infection and Immunity, The University of Melbourne, Parkville, Victoria 3010, Australia.

[3]Infection and Immunity Program and Department of Biochemistry and Molecular Biology, Biomedicine Discovery Institute, Monash University, Clayton, Victoria 3800, Australia.

[4]Department of Biochemistry & Genetics, La Trobe Institute of Molecular Science, La Trobe University, Bundoora, Victoria 3086, Australia.

[5]Australian Research Council Centre of Excellence for Advanced Molecular Imaging, Monash University, Clayton, Victoria 3800, Australia.

[6]Institute of Infection and Immunity, Cardiff University, School of Medicine, Heath Park, Cardiff CF14 4XN, United Kingdom.

* Equally contributing authors. # Jointly supervising authors. Correspondence and requests for materials should be addressed to W.C. (weisan.chen@latrobe.edu.au) or J.R. (jamie.rossjohn@monash.edu).

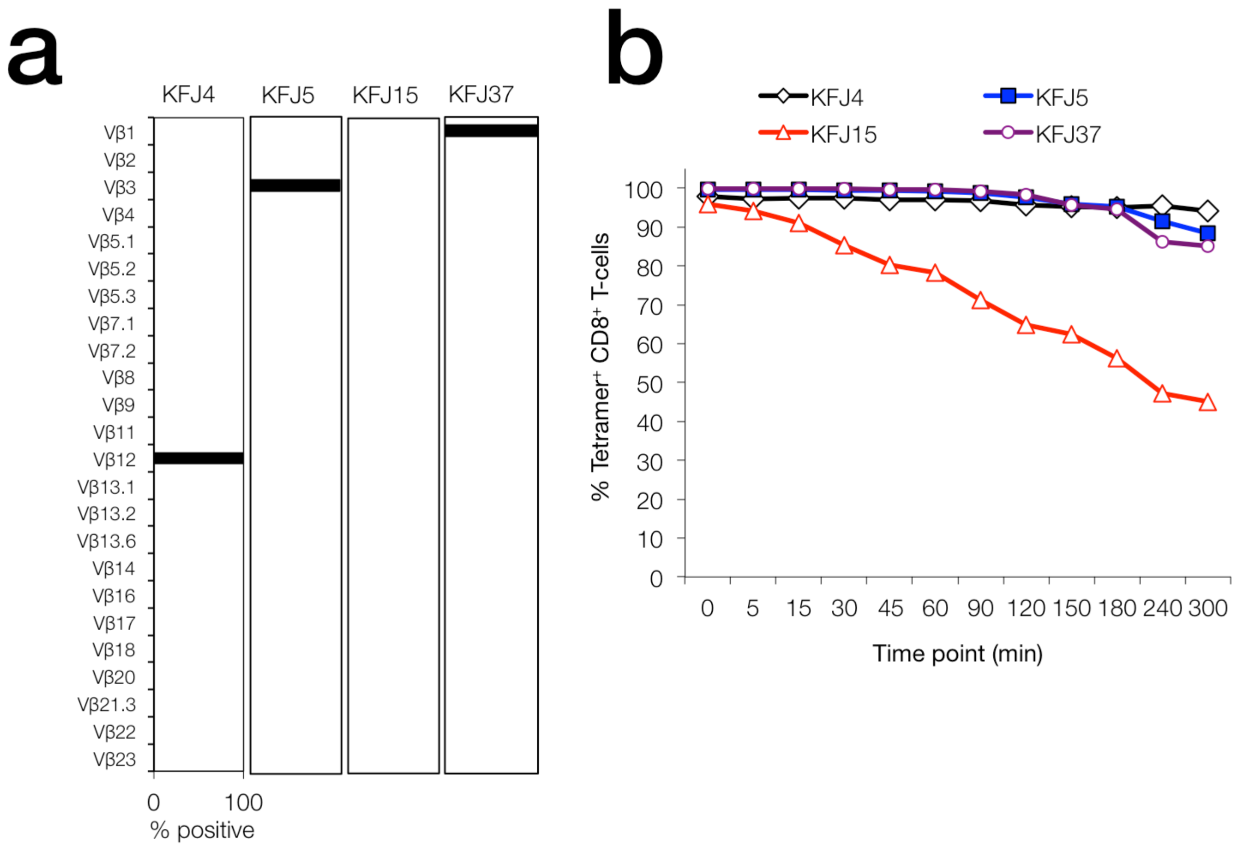

Supplementary Figure 1 | TCR TRBV usages and NY-ESO-1$_{60\text{-}72}$-HLA-B*07:02 tetramer dissociation assay. (**a**) Distinct TCR Vβ usage was found in four NY-ESO-1$_{60\text{-}72}$-HLA-B*07:02-specific CD8^{+} T cell clones. The panel of 24 TCR Vβ-specific monoclonal antibodies used in this study (currently available commercially) does not cover the entire naturally occurring TCR Vβ gene products as the human TCR β locus contains 54 Vβ gene segments. The KFJ15 clone was not stained by any of these TCR Vβ-specific monoclonal antibodies and its TCR Vβ chain usage was subsequently determined by DNA sequencing results. (**b**) NY-ESO-1$_{60\text{-}72}$-HLA-B*07:02 tetramer dissociation assay showed variable avidities of the four CD8^{+} T cell clones. Dissociated tetramer from bound TCR during the dissociation at 37 ^{o}C was 'captured' by the added anti-HLA-B7 monoclonal antibody to prevent tetramer rebinding.

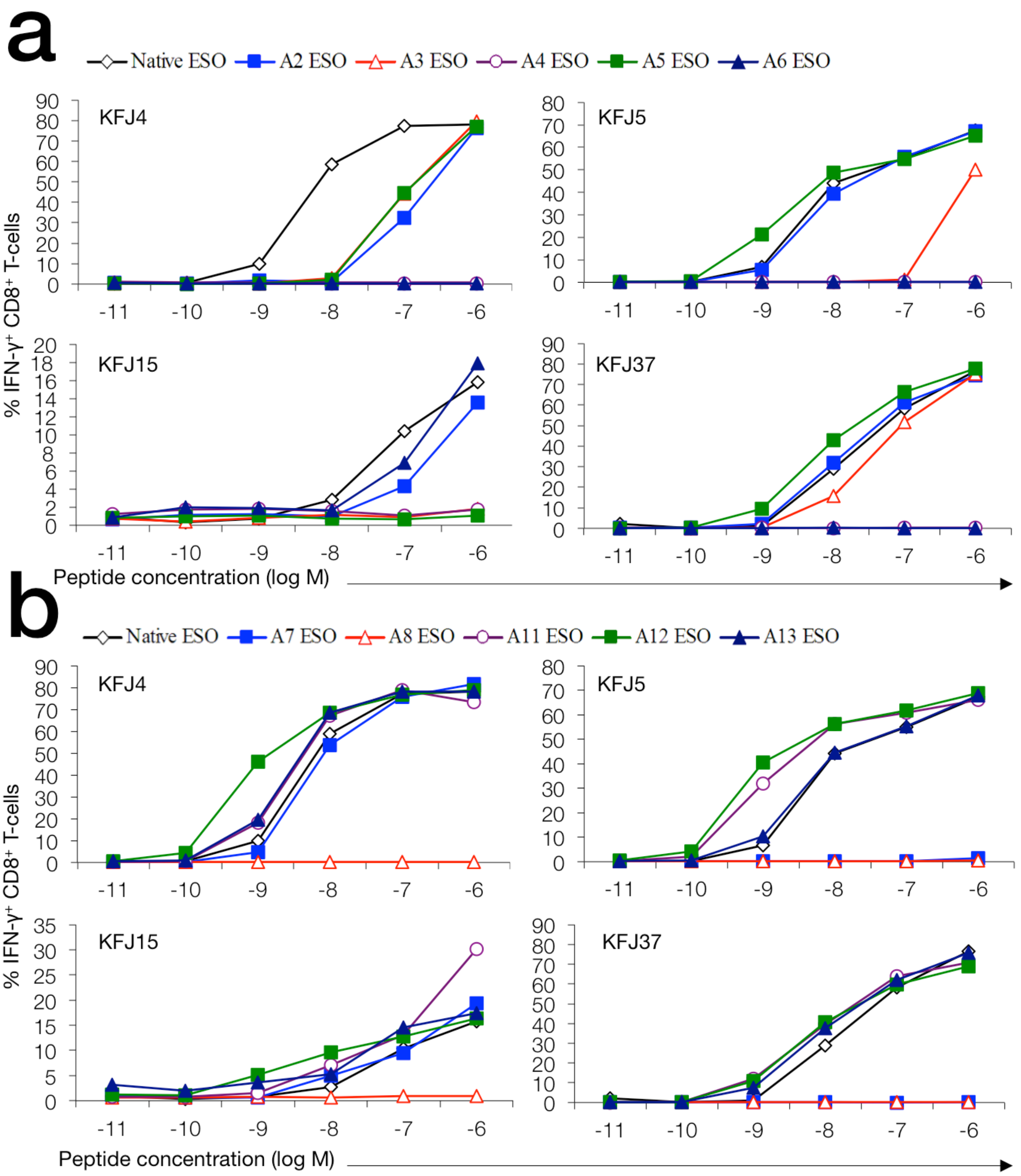

Supplementary Figure 2 | Antigen specificity of KFJ4, KFJ5, KFJ15 and KFJ37 TCRs. Systematic alanine-scanning study revealed different TCR recognition patterns in four NY-ESO-1_{60-72}-HLA-B*0702-specific $CD8^+$ T cell clones. Single alanine substituted NY-ESO-1_{60-72} peptides (A2-A6 ESO) (**a**) and A7-A13 ESO peptides (**b**).

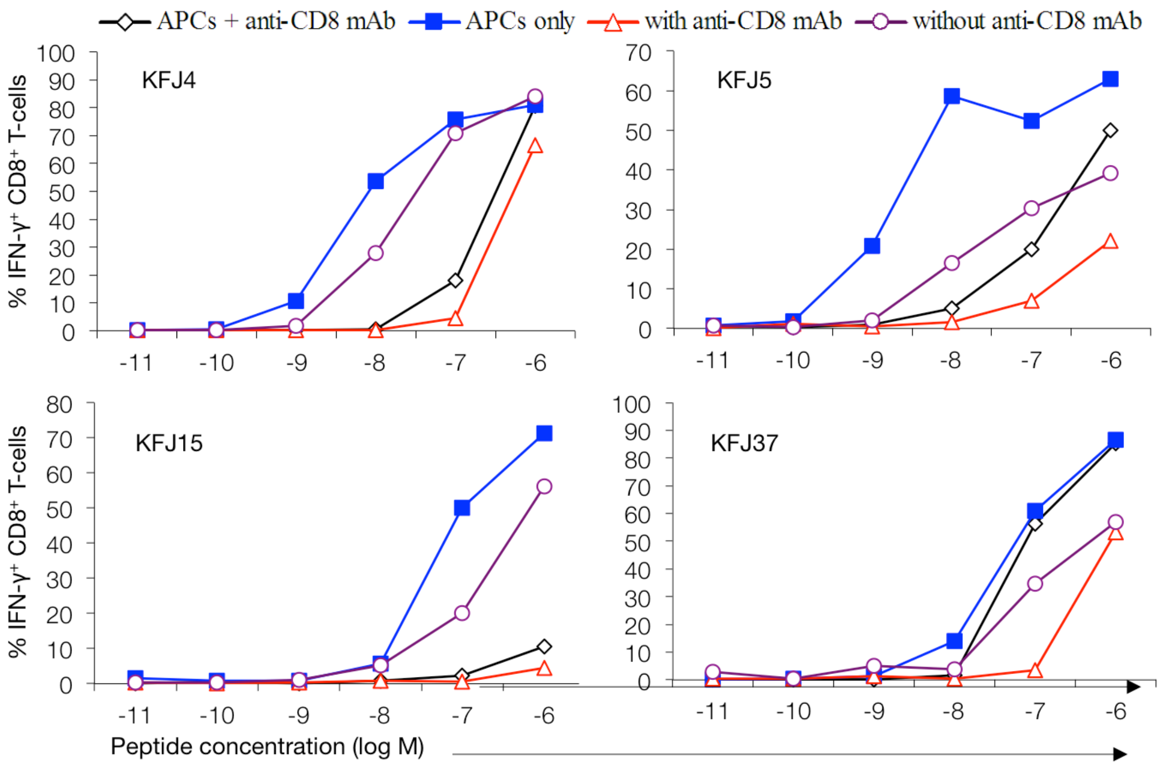

Supplementary Figure 3 | Investigating the differential CD8 co-receptor dependency of the KFJ4, KFJ5, KFJ15 and KFJ37 TCRs during activation. Four different experimental settings were set up to measure the sensitivity of $CD8^+$ T cell clone responses to CD8 co-receptor. CD8 co-receptors of T cells were blocked in the first two experimental groups with anti-CD8 monoclonal antibody. T cells were then stimulated with different NY-ESO-$1_{60\text{-}72}$ peptide concentrations in the presence or absence of HLA-B*07:02^+ antigen-presenting cells (APCs). The next two experimental groups were treated the same way without blocking CD8 co-receptor.

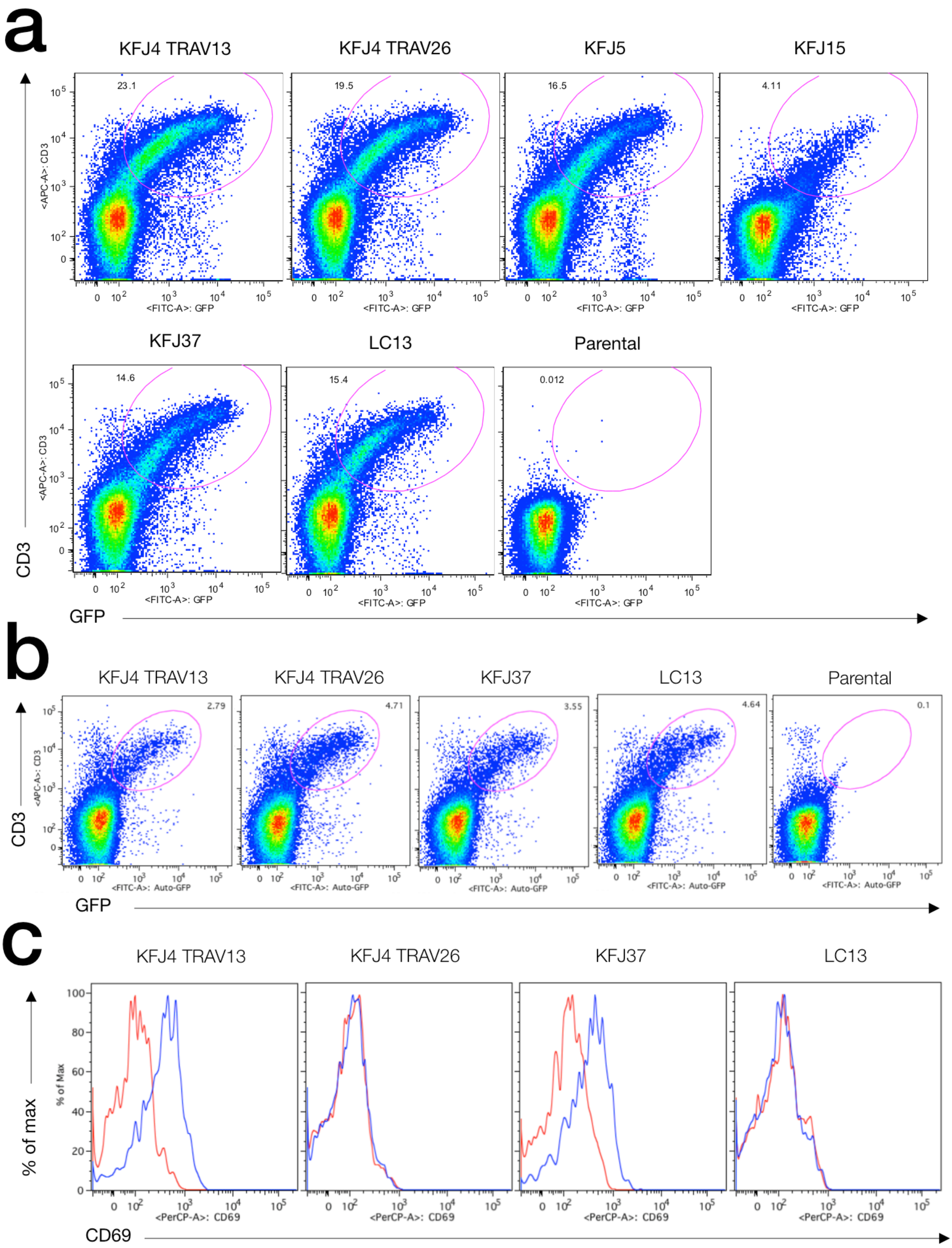

Supplementary Figure 4 | Expression and function of parental and TCR transduced SKW3 cells. (**a**) Cell lines expressing the KFJ4 (TRAV13), KFJ4 (TRAV26), KFJ5, KFJ15,

KFJ37 and LC13 TCRs expressed green fluorescent protein, GFP (a marker gene in the TCR-pMIG expression construct) and CD3 surface receptor. Parental SKW3 cells lacked endogenous TCR α and β chains, and therefore no TCR:CD3 complexes were expressed on the cell surface. LC13, a well-characterised TCR specific to EBNA-3A$_{339\text{-}347}$-HLA-B*08:01,[Ref44] was included as a study control. (**b**) A second independent retroviral transduction experiment was performed for KFJ4 (TRAV13), KFJ4 (TRAV26), KFJ37 and LC13 TCRs, with consistent results obtained for up-regulation of the T cell activation marker CD69 (**c**).

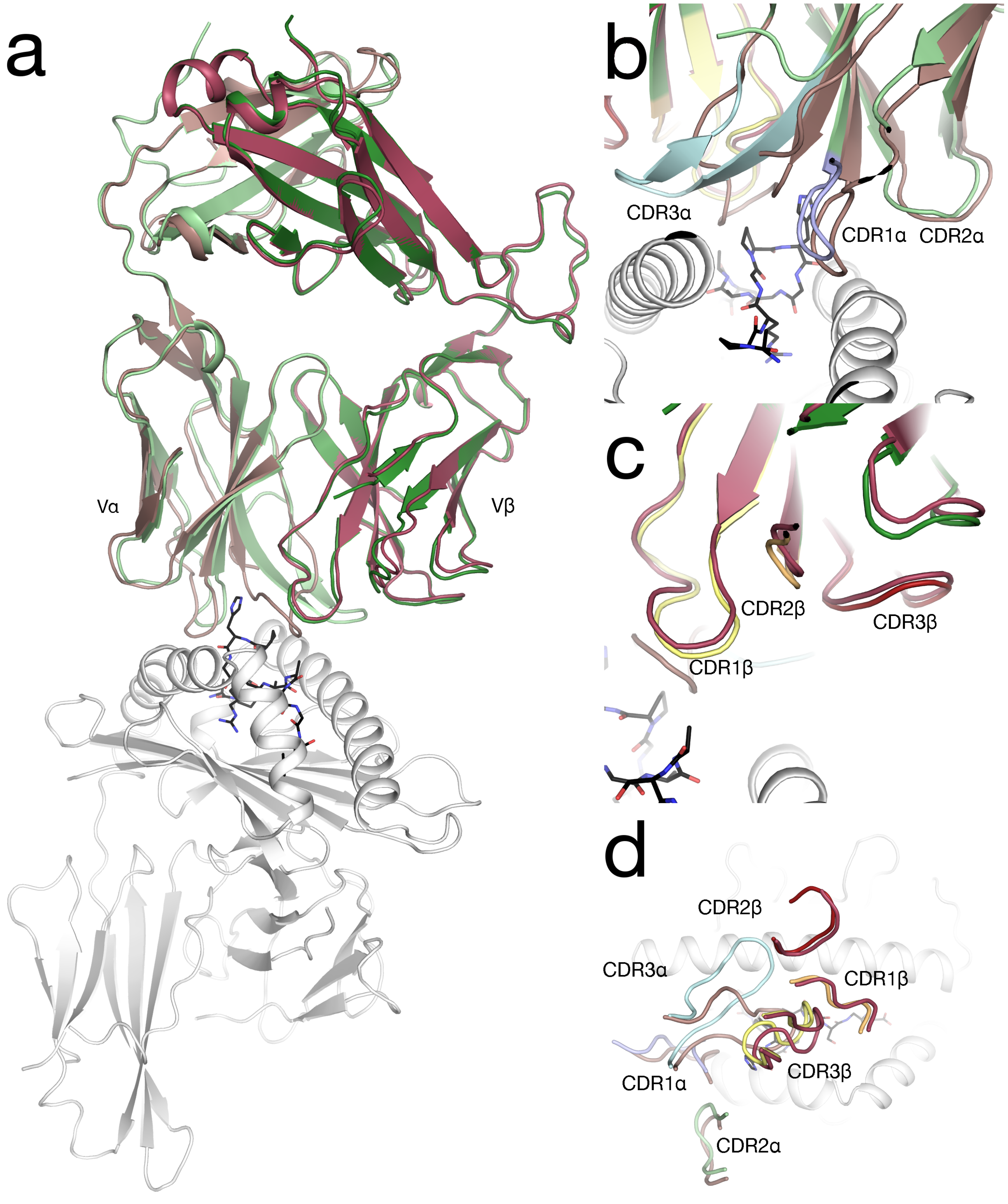

Supplementary Figure 5 | Plasticity of the KFJ5 TCR complementarity determining regions. (**a**) Overview of the KFJ5 TCR-NY-ESO-$1_{60\text{-}72}$-HLA-B*07:02 complex with the KFJ5 TCR binary with the TCR α and β-chains coloured lighter and darker shades of green and red, respectively. The HLA-B heavy chain and β_2-microglobulin molecules are shown as light and dark grey, respectively. Overlay of the CDR α (**b**) and CDRβ (**c**) loops of the KFJ5

TCR show the plasticity of the interface upon NY-ESO-1_{60-72}-HLA-B*07:02 recognition. (**d**) The overview of the TCR interface further highlights the plasticity of the KFJ5 TCR. The complementarity determining region (CDR) loops are coloured as follows; CDR1 α light blue, CDR2 α green, CDR3 α teal, CDR1β orange, CDR2β red, and CDR3β yellow over the HLA-B*07:02 binding groove with the NY-ESO-1_{60-72} peptide shown as black stick

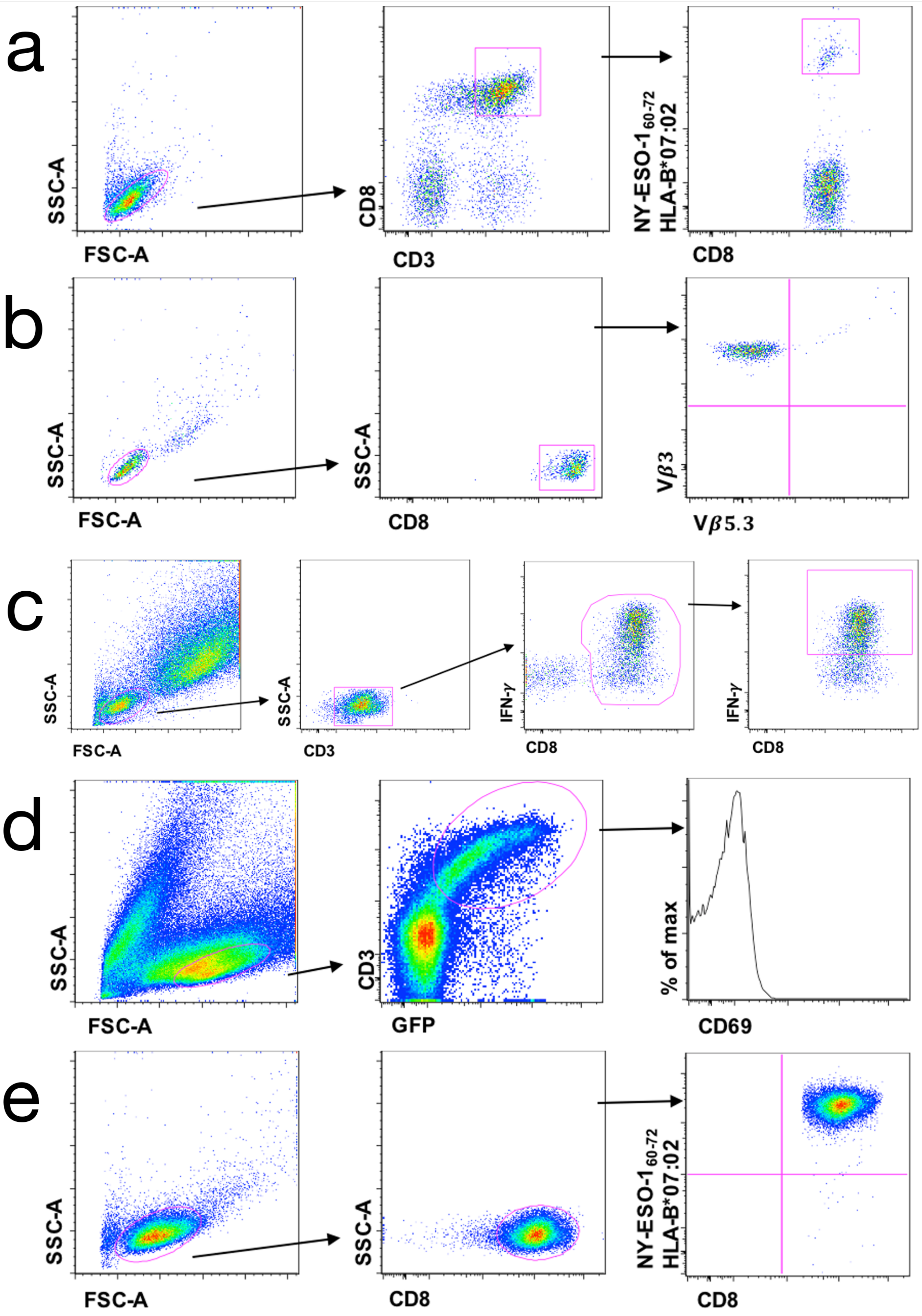

Supplementary Figure 6 | Gating strategies used for all FACS analyses and cell sorting.

(**a**) Gating strategy to sort NY-ESO-$1_{60\text{-}72}$-HLA-B*07:02 tetramer positive $CD8^+$ T cells from *in vitro* NY-ESO-1(55-72) peptide stimulated melanoma patient's PBMC for single cell cloning, as described in methods section. (**b**) Gating strategy to determine the specific TCR $V\beta$ usage in different NY-ESO-$1_{60\text{-}72}$-HLA-B*07:02-specific $CD8^+$ T cell clones as shown in **Supplementary Fig. 1a**. (**c**) Gating strategy to determine the percentage of IFN-γ producing $CD8^+$ T cells following *in vitro* stimulation, as shown in **Fig. 1a, 1e, 1f & 2a** and **Supplementary Fig. 2 & 3**. (**d**) Gating strategy to determine the CD69 up-regulation in TCR transduced SKW3 cells following co-incubation with NY-ESO-$1_{60\text{-}72}$ peptide-pulsed or non-pulsed APC, as shown in **Fig. 1d** and **Supplementary Fig. 4**. (**e**) Gating strategy to determine the percentage of NY-ESO-$1_{60\text{-}72}$-HLA-B*07:02 tetramer positive $CD8^+$ T cells, as shown in **Fig. 1b & 1c** and **Supplementary Fig. 1b**.

Supplementary Table 1 | contacts table between KFJ37 TCR and NY-ESO-1_{60-72}-HLA-B*07:02

	KFJ37 TCR	NY-ESO-1_{60-72}-HLA-B*07:02	Bond Type	NY-ESO-1_{60-72}	Bond Type
CDR1α	Thr30	Glu163, Glu166, Trp167	VDW		
	Asn31-Nδ2, Oδ1	Arg62-NH1, Nε, Trp167-Nε1, Arg62, Glu163, Trp167	H, VDW		
	Tyr33-OH	Ala158-O, Tyr159-N, Glu163-Oε1, Ala158, Tyr159, Glu163	H, VDW	His6-Nε2, His6	H, VDW
FWα	Gln55-Nε2	Gln155	VDW	His6-O	H
CDR2α	Tyr57	Gly162, Glu163	VDW		
	Lys58-Nζ	Glu161-O	H		
CDR3α	Val107			His6, Gly7	VDW
	Asp108-Oδ1, Oδ2, O	Arg62-NH1, NH2, Nε, Arg62	Salt bridge, VDW	His6-N, Gly7-N, His6, Gly7	VDW
	Gln109	Arg62, Gln65, Ile66	VDW		
CDR1β	Leu30	Glu79	VDW		
	Ser31			Ala9	VDW
FWβ	Tyr40-OH			Gly8-O, Gly8, Ala9	H, VDW
CDR2β	Tyr56	Gln75, Glu79	VDW		
	Asn57-Nδ2	Arg78-NH2, Glu79-Oε1, Arg78, Glu79	H, VDW		
	Glu59-Oε1, Oε2	Arg78-NH1, NH2, Nε, Gln75-Nε2, Glu22, Gln75, Arg78	Salt bridge, H, VDW		
	Glu60-O	Gln75-Nε2, Gln75	H, VDW		
FWβ	Arg66-Nε, NH2	Gln75-Oε1, Ala72, Gln75, Thr76	H, VDW		
CDR3β	Ser107-Oγ			Ala9-N, Gly8, Ala9	H, VDW
	Gly108			Ala10, Ala9, Ala10, Ser11	VDW
	His110	Ala153	VDW		
	Ser113-O	Glu155-Oε1, Oε2, Glu155	H, VDW	Pro5-O, Gly8-N, Ala9-N, Pro5, Gly8, Ala9, Ala10	H, VDW
	Asn114	Gln158	VDW	Pro5, His6, Gly7, Gly8, Ala10	VDW
	Glu115			Gly8	VDW
	Gln116-Nε2			Gly7-O, Gly7	H, VDW

Atomic contacts were determined with the CONTACT program from the CCP4i suite.[(REF60)] Hydrogen bonds (H) are defined as 3.5 Å or less. Salt bridge interactions are identified at 4.5 Å or less and van der Waals (VDW) interactions were defined as non-hydrogen bonds at distances of less than 4 Å.

Supplementary Table 2 | contacts table between KFJ5 TCR and NY-ESO-$1_{60\text{-}72}$-HLA-B*07:02

	KFJ5 TCR	NY-ESO-$1_{60\text{-}72}$-HLA-B*07:02	Bond Type	NY-ESO-$1_{60\text{-}72}$	Bond Type
CDR1α	Thr30	Glu163, Glu166, Trp167	VDW		
	Asn31-Oδ1	Trp167-Nε1, Arg62, Glu163, Trp167	H, VDW	Ala1	VDW
	Tyr33	Glu163	VDW	His6	VDW
FWα	Gln55			His6	VDW
CDR2α	Tyr57	Arg62, Glu163	VDW		
CDR3α	Glu108-Oε1, Oε2	Arg62-NH1, NH2, Nε, Arg62	Salt bridge, VDW	Gly4, Pro5	VDW
	Ile109	Ala69	VDW	Pro5	VDW
	Leu110	Ala69	VDW	Pro5	VDW
	Asp111	Gln65, Gln65	VDW		
	Asn112-Oδ2	Gln65-Oε1	H, VDW		
	Phe113	Asp61, Arg62, Gln65	VDW		
CDR3β	Arg109-NH1, NH2	Ala69-O, Thr73-Oγ1, Ala69, Gln72, Thr73	H, VDW	Ala10	VDW
	Gln110-Nε2	Glu152	VDW	Ala10-O, Ala10, Ser11	H, VDW
	Asp113-Oδ2			His6-Nε2, His6	H, VDW

Atomic contacts were determined with the CONTACT program from the CCP4i suite.[REF60] Hydrogen bonds (H) are defined as 3.5 Å or less. Salt bridge interactions are identified at 4.5 Å or less and van der Waals (VDW) interactions were defined as non-hydrogen bonds at distances of less than 4 Å.

Supplementary Table 3 | Monoclonal antibodies

Antibody target	Clone name	Source	Dilution used	Host
Anti-human CD3	UCHT1	BD Biosciences	1:50	mouse
Anti-human CD8	RPA-T8	BD Biosciences	1:50	mouse
Anti-human CD69	FN50	BD Biosciences	1:50	mouse
Anti-human IFN-γ	4S.B3	BD Biosciences	1:100	mouse
Anti-human TCR Vβ1	BL37.2	Beckman Coulter	1:20	rat
Anti-human TCR Vβ2	MPB2D5	Beckman Coulter	1:20	mouse
Anti-human TCR Vβ3	CH92	Beckman Coulter	1:20	mouse
Anti-human TCR Vβ4	WJF24	Beckman Coulter	1:20	rat
Anti-human TCR Vβ5.1	IMMU157	Beckman Coulter	1:20	mouse
Anti-human TCR Vβ5.2	36213	Beckman Coulter	1:20	mouse
Anti-human TCR Vβ5.3	3D11	Beckman Coulter	1:20	mouse
Anti-human TCR Vβ7.1	ZOE	Beckman Coulter	1:20	mouse
Anti-human TCR Vβ7.2	ZIZOU4	Beckman Coulter	1:20	mouse
Anti-human TCR Vβ8	56C5.2	Beckman Coulter	1:20	mouse
Anti-human TCR Vβ9	FIN9	Beckman Coulter	1:20	mouse
Anti-human TCR Vβ11	C21	Beckman Coulter	1:20	mouse
Anti-human TCR Vβ12	VER2.32	Beckman Coulter	1:20	mouse
Anti-human TCR Vβ13.1	IMMU222	Beckman Coulter	1:20	mouse
Anti-human TCR Vβ13.2	H132	Beckman Coulter	1:20	mouse
Anti-human TCR Vβ13.6	JU74.3	Beckman Coulter	1:20	mouse
Anti-human TCR Vβ14	CAS1.1.3	Beckman Coulter	1:20	mouse
Anti-human TCR Vβ16	TAMAYA1.2	Beckman Coulter	1:20	mouse
Anti-human TCR Vβ17	E17.5F3	Beckman Coulter	1:20	mouse
Anti-human TCR Vβ18	BA62.6	Beckman Coulter	1:20	mouse
Anti-human TCR Vβ20	ELL1.4	Beckman Coulter	1:20	mouse
Anti-human TCR Vβ21.3	IG125	Beckman Coulter	1:20	mouse
Anti-human TCR Vβ22	IMMU546	Beckman Coulter	1:20	mouse
Anti-human TCR Vβ23	AF23	Beckman Coulter	1:20	mouse

All monoclonal antibody staining was performed in 50 μL of PBS
